# Mode of Delivery according to Leisure Time Physical Activity before and during Pregnancy: A Multicenter Cohort Study of Low-Risk Women

**DOI:** 10.1155/2017/6209605

**Published:** 2017-03-13

**Authors:** Emilie Nor Nielsen, Per Kragh Andersen, Hanne Kristine Hegaard, Mette Juhl

**Affiliations:** ^1^The Research Unit Women's and Children's Health, The Juliane Marie Centre, Copenhagen University Hospital, Rigshospitalet, Dep. 7821, Blegdamsvej 9, 2100 Copenhagen, Denmark; ^2^Section of Biostatistics, Department of Public Health, Øster Farimagsgade 5 opg. B, P.O. Box 2099, 1014 Copenhagen K, Denmark; ^3^Department of Obstetrics, Copenhagen University Hospital, Rigshospitalet, Copenhagen, Denmark; ^4^The Institute of Clinical Medicine, Faculty of Health and Medical Sciences, University of Copenhagen, Blegdamsvej 3, Copenhagen, Denmark; ^5^Midwifery Department, Metropolitan University College, Sigurdsgade 26, 2200 Copenhagen, Denmark; ^6^Department of Public Health, Øster Farimagsgade 5, 1014 Copenhagen K, Denmark

## Abstract

*Objectives*. To examine the association between maternal leisure time physical activity and mode of delivery.* Study Design*. Population-based multicentre cohort. From the Danish Dystocia Study, we included 2,435 nulliparous women, who delivered a singleton infant in cephalic presentation at term after spontaneous onset of labor in 2004-2005. We analysed mode of delivery according to self-reported physical activity at four stages, that is, the year before pregnancy and during first, second, and third trimester, in logistic regression models. Further, we combined physical activity measures at all four stages in one variable for a proportional odds model for cumulative logits.* Main Outcome Measures*. Mode of delivery (emergency caesarean section; vacuum extractor; spontaneous vaginal delivery).* Results*. The odds of emergency caesarean section decreased with increasing levels of physical activity with statistically significant trends at all four time stages except the third trimester. This tendency was confirmed in the proportional odds model showing 28% higher odds of a more complicated mode of delivery among women with a low activity level compared to moderately active women.* Conclusions*. We found increasing leisure time physical activity before and during pregnancy associated with a less complicated delivery among low-risk, nulliparous women.

## 1. Introduction

Physical activity during pregnancy is associated with a reduced risk of preterm delivery, gestational diabetes, and possibly also preeclampsia [1–7] but has also been associated with an increased risk of miscarriage among women who exercised early in pregnancy [8]. Few studies have examined physical activity before pregnancy, but associations have been reported with a reduced risk of gestational diabetes and preeclampsia [9]. Due to the general benefits of physical activity on mortality and physical and mental health [10] regular physical activity during pregnancy is recommended in Denmark and other countries since 2002 [11–15]. Even so, the results on how, or if, physical activity affects the course of delivery, are inconclusive [16–26]. Hence, one meta-analysis, based on 4 randomized controlled trials, reported no association between physical activity and caesarean section (C-section) [16], while another one, based on 16 randomized controlled trials, found structured physical exercise during pregnancy to be associated with a reduced risk of C-section [5], which was also the conclusion of a recent meta-analysis including 8 studies on normal-weight women [26]. Finally, a meta-analysis from 2015 suggested that regular exercise during pregnancy was modestly associated with increased chance of normal delivery; the authors, however, stressed the need for further research, including measures of the intensity and the gestational timing of physical exercise [21]. Large population-based studies are sparse and also report inconclusive findings [27–29].

In clinical obstetrics, vaginal deliveries are usually preferred to C-sections in low-risk pregnant and laboring women, because C-sections have been associated with an increased risk of complications to anaesthesia, excessive blood loss, respiratory complications, longer recovery periods in the mother, risks associated with C-section antea in subsequent pregnancies (e.g., placenta previa, placenta accrete, and stillbirth), iatrogenic prematurity, respiratory complications, and referral to neonatal unit in the child, and also long-term effects in the child, such as asthma, systematic connective tissue disorders, juvenile arthritis, inflammatory bowel diseases, immune deficiencies, and leukaemia, have been suggested [30–34].

Even though there have been several original studies and also meta-analyses summing up results, findings are still inconclusive, and previous research generally lacks detailed information on exercise (such as measures from the prepregnancy period and the first trimester of pregnancy) and on obstetric data related to mode of delivery (such as instrumental vaginal delivery, primary versus secondary C-section, and elective versus emergency C-section). These are relevant factors, since the general risk of C-section is dependent on parity, the risk of C-section is substantially increased after a previous C-section, and aetiology and risk of complications related to the C-section surgery varies largely between elective and emergency C-sections. Finally, since instrumental vaginal delivery and emergency C-section can be considered a continuum (away from an uncomplicated, vaginal delivery), vacuum extractor should ideally also be included in studies on mode of delivery.

The aim of this study was to examine the association between leisure time physical activity the year before pregnancy and during pregnancy and mode of delivery. We hypothesize that regular physical activity reduces the incidence of emergency C-section and the use of vacuum extractor.

## 2. Materials and Methods

### 2.1. Study Population

We used data from the Danish Dystocia Study, a population-based multicentre study with prospectively collected data from 9 obstetrics departments in Denmark during 2004-2005. The study included women in Robson delivery Group 1, that is, women with a singleton vertex infant and spontaneous onset of labor at ≥37 completed gestational weeks [35, 36]. Typical reasons for never entering Robson Group 1 include, for example, preterm delivery, induced delivery, breech presentation, or planned C-section. In addition, participants should be 18 years or older and able to read and understand Danish language. Recruitment took place in antenatal clinics at 33 gestational weeks, the women gave written informed consent, and a self-reported baseline questionnaire was administered at 37 gestational weeks.

A number of 2652 women fulfilled the criteria and had completed the baseline questionnaire. From this number, we excluded women with missing data on mode of delivery (*n* = 22), physical activity before pregnancy (*n* = 32), physical activity during first (*n* = 3), second (*n* = 3) and third trimester (*n* = 12), age (*n* = 5), educational level (*n* = 19), smoking during pregnancy (*n* = 10), and prepregnancy body mass index (*n* = 111), resulting in a final study population of 2435 women.

Permission to establish the database was obtained from the Danish Data Protection Agency j.no. 2004-41-4545. Since no invasive procedures were applied in the study, no Ethics Committee System approval was required by Danish law. The policy of the Helsinki Declaration was followed throughout the data collection and analyses.

### 2.2. Measurement of Physical Activity

In the Danish Dystocia Study baseline questionnaire at gestational week 37, the women were asked about physical activity level at each of four stages (the year before pregnancy/first trimester/second trimester/third trimester). Physical activity was analysed using a four-item physical activity score [37]: “When you look back on (e.g., the year before your current pregnancy), which would you say is the most appropriate description of your activities?” #1 Hard training and competing sports regularly and several times a week (“competitive sports”), #2 sports or heavy gardening at least four hours a week (“moderate-to-heavy physical activity”), #3 walking, cycling, or other light exercises at least four hours a week (including Sunday walks, light gardening, and cycling/walking to work) (“light physical activity”), or #4 reading, watching television, or pursuing some other sedentary occupation (“sedentary lifestyle”). For additional analyses, we assigned a score to each of the four activity levels and constructed an activity sum score by adding the values of the four stages resulting in one single score. The sum score summarized each woman's physical activity level as a number between 4 and 16. This quantitative measure was further categorized into high (<11), moderate (11-12), and low (>12) physical activity level. The cut points at 10/11 and 12/13 were chosen to obtain similar group sizes for the two extreme exposure groups. For the low physical activity level, the cut-off point at 12/13 implied that the woman had reported sedentary lifestyle (#4) in at least one of the four stages.

### 2.3. Measurement of Other Covariates

Self-reported data on age, educational level (as the best available measure of socioeconomic status), prepregnancy body mass index, smoking, and physical working conditions came from the baseline questionnaire in gestational week 37 with categorization as displayed in Table 1. We chose covariates to be included in the model a priori, with the exception of physical working conditions. This variable was not included in the final model due to a substantial number of missing values (32%). However, we did perform a sensitivity analysis. Body mass index and smoking were considered potential effect modifiers. The biochemical effect of physical activity varies according to both factors; body mass index is associated with mode of delivery, and smoking is associated with suboptimal placental function and fetal growth retardation and, thus, associated with an increased risk of fetal asphyxia during delivery and mode of delivery [38–42].

### 2.4. Measurement of Mode of Delivery

Data on mode of delivery were collected from records completed by midwives in connection with the delivery and registered as spontaneous vaginal delivery, vacuum extraction, or emergency C-section; emergency C-section is defined as any nonelective C-section. In the following, “any vaginal delivery” refers to spontaneous vaginal deliveries and vacuum extractor assisted deliveries together.

### 2.5. Statistical Analysis

We performed *χ*^2^ test for independence between maternal characteristics and physical activity the year before pregnancy. We calculated odds ratios and *p* values for trend for emergency C-section (versus any vaginal delivery) and for emergency C-section and vacuum extractor (versus spontaneous delivery) according to level of physical activity the year before pregnancy and in the first, second, and third trimester. This approach comprises a high degree of detail (four categorical exposure variables and two outcome measures). In order to confirm, or not confirm, findings from this straight-forward approach, physical activity was also analyzed using a more condensed model. Hence, we used a proportional odds model [43] to examine the association between an activity sum score and mode of delivery as an ordinal outcome, and we calculated odds ratios for going one step in the direction of a more complicated mode of delivery. The use of the activity sum score was evaluated in several steps. First, we tested for linearity for each of the individual physical activity scores, next, we tested whether all of the individual physical activity scores had the same association with the ordinal outcome, and finally the proportional odds assumption was tested. Since no significant violations of these assumptions were identified, the tests are not reported in what follows. Finally, we tested for no interaction between physical activity and maternal smoking and between physical activity and prepregnancy body mass index. Only adjusted estimates are presented, because the inclusion of adjustment variables showed almost identical results as in the crude analyses. Analyses were carried out in SAS Statistical Software V9.1.

## 3. Results

Table 1 shows the reported activity level in each of four time phases. Few women engaged in competitive sport, and, overall, the proportion of women, who reported moderate-to-heavy physical activity or competitive sport, was markedly reduced from before pregnancy to first trimester, and further gradually reduced throughout pregnancy (Table 1). Likewise, a substantial rise in women with a sedentary lifestyle was seen over the time span.

Table 2 shows leisure time physical activity the year before pregnancy and mode of delivery according to maternal baseline characteristics. Overall, 95 percent of the women were physically active at some level the year before pregnancy, and 76 percent had a spontaneous vaginal delivery. Women with a shorter education tended to be more sedentary, as did obese women, smokers, and women with a less physically demanding job. As for mode of delivery, the likelihood of emergency C-section was higher among women, who were older, who had no or a short education, who were overweight or obese, and who were light smokers or did not smoke at all.

Table 3 shows reduced odds for emergency C-section (versus any vaginal delivery) among women with some degree of physical activity before or during pregnancy. This tendency, compromised by limited statistical power, was confirmed by statistically significant *p* values for trend between physical activity before pregnancy and during first and second trimester and complicated delivery (Table 3). When collapsed into one measure, odds for a complicated delivery showed a fairly similar pattern, that is, reduced odds for emergency C-section and vacuum extractor (versus spontaneous vaginal delivery) among women with some degree of physical activity.

Table 4 shows the odds ratios for moving one step on the ordinal outcome scale in the direction of a more complicated mode of delivery, that is, the step from spontaneous vaginal delivery to vacuum extractor and emergency C-section or the step from any vaginal delivery to emergency C-section, according to a sum score of physical activity covering all four stages. The association between the sum score and mode of delivery was consistent with the initial analyses as presented in Table 3; for example, women in the higher activity group had lower odds of a more complicated delivery than women, who were moderately physically active, and those who were moderately active had lower odds than women with the lowest activity level. We tested for and found no interaction between physical activity and smoking or prepregnancy body mass index. Also, adding a variable on working conditions (physically demanding: yes/no) in the proportional odds model did not change the effect of physical activity as a sum score (complete case analysis, *N* = 1769) (data not shown).

## 4. Discussion

Among 2,435 nulliparous women with expected uncomplicated delivery, physically active women were less likely to have a complicated delivery than physically inactive women. The results were robust over different levels of physical activity; the higher the level, the lower the odds of emergency C-section (versus any vaginal delivery) and of emergency C-section and vacuum extractor (versus spontaneous delivery), and they were robust over different pregestational and gestational time phases of exposure; that is, we saw the same tendencies for the year preceding pregnancy and the three trimesters of pregnancy.

Our findings are in agreement with some previous observational findings but not with others [5, 21, 27–29, 44, 45], and in disagreement with a randomized controlled trial by Barakat et al. [46]. Barakat et al. study included 142 women of low to middle socioeconomic position, and it may be expected that women who agree to randomization of their lifestyle during pregnancy constitute a selected group. Further, some of the observational studies included both elective and emergency C-section [27, 45, 47]; in the present study elective C-sections were excluded. This reduces comparability with part of the existing literature. However, our results are less likely to be biased by indications for the elective C-section.

Even though our data indicated a consistent trend between increasing levels of physical activity and less complicated deliveries, part of our findings may be explained by a “healthy exerciser effect,” that is, the effect of confounding by indication. If women with poor health in pregnancy are less likely to exercise than healthier women, and, at the same time, some risk factors for poor health are shared with those for C-section, then physical activity will turn out as a preventive factor for C-section as a consequence of a lower generic risk among the physically active women. Moreover, epidemiological cohort studies like the Danish Dystocia Study usually have participants that are on average healthier and more socioeconomically advanced than the background population. We do not believe this, however, to cause selection bias regarding a possible causal association between exercise and mode of delivery.

Among Danish pregnant women associations between background factors such as increasing age, higher educational level, normal prepregnancy body mass index, and nonsmoking and leisure time physical activity during pregnancy have been found [48]. In the present study, however, adjustment for these factors did not alter the association between leisure time physical activity and a less complicated mode of delivery substantially.

Assessment of physical activity in this study relied on self-reporting, and, to some degree, recall of physical activity level, which may imply information bias, but data on physical activity was collected before mode of delivery was known, and possible misclassification should therefore not be differential. The questions developed by Saltin et al. have been found valid for self-reported physical activity in epidemiological studies [37, 49].

This study concentrated on physical activity during leisure time, and thus we did not intend to evaluate physical activity as a whole. Different mechanisms seem to be in play for physical activity during leisure time and at work; roughly spoken, occupational-related physical activity tend to be associated with adverse pregnancy/birth outcomes and recreational activity with healthy outcomes [50]. Although the questions by Saltin on physical activity were probably intended to measure leisure time physical activity, the term “leisure time” is not specified to the respondents, and, thus, some women may have included work-related activities or household activities, and others not. Household chores seem to contribute substantially to the total amount of physical activity performed by women in the child-bearing ages [50–53]. We find it unlikely, though, that work related activity was included by the women, because the wording of the four categories clearly points towards leisure time activities. The questionnaire was filled in at gestational age 37, at which point in time almost all pregnant women holding a job in Denmark are on maternity leave due to social rights, which diminishes the potential problem of including work-related physical activity in the third trimester. We did not include information on physical working conditions in the main analyses because of a substantial number of missing. We believe that the main reasons for not answering work-related questions were that the women did not hold a job or had already ceased working due to maternity leave. Sensitivity analyses restricted to women with information on working conditions did not change the conclusions.

By using a homogeneous study population (i.e., Robson Group 1 deliveries) we should have reduced the extent of confounding by some of the risk factors for emergency C-section. We consider this group of nulliparous women with no indication of induction of labor or elective C-section well suited for this study, as we expect only few, if any, of these women had been advised against physical activity during pregnancy. Our study population may theoretically comprise women with moderate hypertension and possibly also moderate preeclampsia (that is not severe enough to cause, e.g., induction of labor). We believe, however, that hypertension/preeclampsia that is considered not severe at the time of delivery is unlikely to have been present before pregnancy or earlier in pregnancy at a level that would cause restrictive recommendations regarding physical activity. In this study, we wished to examine if, and how, physical activity is associated with mode of delivery among a group of women with expectedly uncomplicated deliveries, that is, pregnancies that have not been classified as complicated to a degree that have caused induction, C-section, or other interventions until the time of spontaneous labor at term. This correlates with the original purpose of the mother-study, where participants were included in the Danish Dystocia Study only if they were nulliparous, had a single cephalic fetal presentation, and had spontaneous onset of labor at ≥37 gestational weeks, according to the Robson Classification, Group 1. The Robson classification is widely used in clinical practice (as well as in research) to audit and monitor the quality of antenatal and perinatal services, and by sticking to this definition of the study population, our results reflects daily clinical practice and debates and enhances comparability with other research.

Our findings correlate with previous results from the Danish Dystocia Study, where athletics or heavy gardening > or =4 h per week was found protective for labor dystocia [54]. Should our findings reflect causality, this may be explained by larger and better functioning placentae; Jackson and colleagues found physical exercise in first part of pregnancy associated with increased placental vascular volume and villi, with further increase after exercise throughout pregnancy [55]. Regular aerobic exercise during pregnancy is associated with improved physical fitness [20], and higher maximal oxygen uptake has been found associated with shorter duration of labor [56]. If leisure time physical activity reduces the risk of a complicated delivery, this is not only of clinical and public health relevance but may also have economic implications. Bungum et al. estimated that one-third of C-sections might be attributed to a sedentary behaviour and that medical expenses could have been substantially reduced, had these women not been sedentary [44].

## 5. Conclusions

In this population of low-risk, nulliparous women we found an increasing level of leisure time physical activity associated with a less complicated mode of delivery, that is, a reduced risk of emergency C-section, when compared to any vaginal delivery and a reduced risk of a complicated delivery including vacuum extraction or emergency C-section compared to a spontaneous vaginal delivery. This study included detailed measures of the timing of physical activity, including the prepregnancy period, which has only been sparsely reported so far. Findings from the study suggest that leisure time physical activity may play a role in reducing the number of emergency C-section and assisted vaginal deliveries, which is of public health interest, since physical activity is inexpensive and produces few negative side effects.

## Figures and Tables

**Table 1:** Physical activity levels the year before pregnancy and during first, second, and third trimester. The Danish Dystocia Study, 2004-2005. *N* = 2435.

Physical activity	Before pregnancy	First trimester	Second trimester	Third trimester
	*N* (%)	*N* (%)	*N* (%)	*N* (%)
Sedentary	131 (5.4)	289 (12)	309 (13)	700 (29)
Light	1566 (64)	1728 (71)	1890 (78)	1651 (68)
Moderate-to-heavy	636 (26)	370 (15)	225 (9.2)	81 (3.3)
Competitive sport	102 (4.2)	48 (2.0)	11 (0.5)	3 (0.1)

**Table 2:** Physical activity levels the year before pregnancy and mode of delivery according to maternal characteristics. The Danish Dystocia Study, 2004-2005. *N* = 2435.

Characteristics	Physical activity the year before pregnancy		Mode of delivery		
	Any physical activity	Sedentary	Spontaneous vaginal delivery	Vacuum extractor	Emergency C-section
Total	2304 (94.6)	131 (5.4)	1854 (76.1)	369 (15.2)	212 (8.7)

Maternal age (years)					
<25	375 (96.2)	15 (3.9)	317 (81.3)	42 (10.8)	31 (8.0)
25–29	1132 (93.9)	73 (6.1)	935 (77.6)	177 (14.7)	93 (7.7)
30–34	635 (94.4)	38 (5.7)	480 (71.3)	126 (18.7)	67 (10.0)
≥35	162 (97.0)	5 (3.0)	122 (73.1)	24 (14.4)	21 (12.6)
Educational level after secondary education					
<3 years	781 (92.5)	63 (7.5)	619 (73.3)	144 (17.1)	81 (9.6)
3-4 years	731 (95.9)	31 (4.1)	587 (77.0)	102 (13.4)	73 (9.6)
>4 years	361 (96.0)	15 (4.0)	282 (75.0)	69 (18.4)	25 (6.7)
Never commenced/interrupted training studying	177 (94.7)	10 (5.4)	141 (75.4)	26 (13.9)	20 (10.7)
	254 (95.5)	12 (4.5)	225 (84.6)	28 (10.5)	13 (4.9)
Body mass index					
<18.5	107 (98.2)	2 (1.8)	90 (82.6)	15 (13.8)	4 (3.7)
18.5–24.99	1685 (95.3)	8 (4.8)	1357 (76.7)	268 (15.2)	144 (8.1)
25–30	374 (95.9)	16 (4.1)	288 (73.9)	60 (15.4)	42 (10.8)
>30	138 (82.6)	29 (17.4)	119 (71.2)	26 (15.6)	22 (13.2)
Smoking (cigarettes)					
No smoking	2085 (95.1)	107 (4.9)	1675 (76.4)	331 (15.1)	186 (8.5)
1–10 per day	165 (93.2)	12 (6.8)	126 (71.2)	28 (15.8)	23 (13.0)
11–20 per day	54 (81.8)	12 (18.2)	53 (80.3)	10 (15.2)	3 (4.6)
Working conditions^a^					
Physically demanding	791 (97.1)	24 (2.9)	602 (73.9)	129 (15.8)	84 (10.3)
Not physically demanding	891 (93.4)	63 (6.6)	733 (76.8)	141 (14.8)	80 (8.4)

^a^Missing = 666.

C-section = caesarean section.

**Table 3:** Adjusted odds ratios for mode of delivery according to leisure time physical activity before and during pregnancy. The Danish Dystocia Study. *N* = 2435.

Physical activity	Emergency C-section (versus any vaginal delivery)			Emergency C-section and vacuum extractor (versus spontaneous vaginal delivery)		
	OR^a^	95% CI	*p* value for trend	OR^a^	95% CI	*p* value for trend
Before pregnancy						
Sedentary	1	—	0.0372	1	—	0.1043
Light	0.63	0.37–1.08		0.80	0.54–1.20	
Moderate-to-heavy	0.57	0.32–1.04		0.73	0.47–1.12	
Competitive sport	0.28	0.09–0.88		0.63	0.34–1.19	
First trimester						
Sedentary	1	—	0.0301	1	—	0.0083
Light	0.89	0.58–1.34		0.77	0.58–1.02	
Moderate-to-heavy	0.57	0.32–1.03		0.60	0.41–0.86	
Competitive sport	0.39	0.09–1.67		0.65	0.31–1.37	
Second trimester						
Sedentary	1	—	0.0303	1	—	0.0070
Light	0.73	0.50–1.08		0.80	0.61–1.05	
Moderate-to-heavy	0.47	0.23–0.93		0.56	0.36–0.85	
Competitive sport	0.82	0.10–6.92		0.53	0.11–2.53	
Third trimester						
Sedentary	1	—	0.1466	1	—	0.0134
Light	0.80	0.59–1.09		0.80	0.65–0.99	
Moderate-to-heavy	0.77	0.32–1.85		0.63	0.35–1.13	
Competitive sport	—	—		—	—	

^a^Adjusted for maternal age, educational level when secondary education is completed, smoking, and prepregnancy body mass index.

C-section = caesarean section, OR = odds ratio, and CI = confidence interval.

**Table 4:** Adjusted odds ratios for a more complicated delivery, that is, emergency C-section (versus any vaginal delivery) or emergency C-section and vacuum extractor (versus spontaneous vaginal delivery) according to a physical activity sum score covering the time period from one year before pregnancy and all three pregnancy trimesters. The Danish Dystocia Study. *N* = 2435.

Physical activity sum score	OR^a^	95% CI	*p* value for trend
Low activity level	1.28	1.04–1.58	0.0029
Moderate activity level	1	—	
High activity level	0.77	0.58–1.02	

^a^Adjusted for maternal age, educational level when secondary education is completed, smoking, and prepregnancy body mass index.

C-section = caesarean section, OR = odds ratio, and CI = confidence interval.
